# Nomogram prediction model for length of hospital stay following laparoscopic appendectomy in pediatric patients: a retrospective study

**DOI:** 10.3389/fped.2024.1441263

**Published:** 2024-12-13

**Authors:** Ming Liu, Ping Yang, Yunpeng Gou, Qiang Chen, Dong Xu

**Affiliations:** Department of Pediatric Surgery, Suining Central Hospital, Suining, Sichuan Province, China

**Keywords:** length of stay, children, appendicitis, predictors, nomogram

## Abstract

**Objective:**

The aim of this research was to develop and internally validate a nomogram for forecasting the length of hospital stay following laparoscopic appendectomy in pediatric patients diagnosed with appendicitis.

**Methods:**

We developed a prediction model based on a training dataset of 415 pediatric patients with appendicitis, and hospitalization data were collected retrospectively from January 2021 and December 2022. The primary outcome measure in this study was hospital length of stay (LOS), with prolonged LOS defined as admission for a duration equal to or exceeding the 75th percentile of LOS, including the discharge day. Risk factor analysis was conducted through univariate and multivariate logistic regression analyses. Based on the regression coefficients, a nomogram prediction model was developed. The discriminative performance of the predicting model was evaluated using the C-index, and an adjusted C-index was computed through bootstrapping validation. Calibration curves were generated to assess the accuracy of the nomogram. Decision curve analysis was conducted to determine the clinical utility of the predicting model.

**Results:**

Predictors contained in the prediction nomogram included Age, neutrophil-to-lymphocyte ratio, C-reactive protein level, operative time, appendiceal fecalith, and drainage tube. The C-index of the prediction nomogram was determined to be 0.873 (95% CI: 0.838–0.908), with a corrected C-index of 0.8625 obtained through bootstrapping validation (1,000 resamples), indicating the model's favorable discrimination. Calibration curves illustrated a strong agreement between predicted and observed outcomes. According to the decision curve analysis of the nomogram, the predictive model demonstrates a net benefit at threshold probabilities exceeding 2%.

**Conclusion:**

This nomogram, incorporating variables such as Age, neutrophil-to-lymphocyte ratio, C-reactive protein level, operative time, appendiceal fecalith, and drainage tube, offers a convenient method for assessing the duration of hospitalization in pediatric patients with appendicitis.

## 1 Introduction

Acute appendicitis is a prevalent acute abdominal disease in children, with an estimated annual incidence of 83 per 100,000 individuals (1). Currently, laparoscopic appendectomy is the primary treatment method for acute appendicitis in children due to its rapid recovery time and reduced postoperative pain (2–5). Complicated appendicitis, characterized by gangrene and perforation, is associated with prolonged hospital stays, increased costs, higher rates of complications, and a poorer overall prognosis compared to uncomplicated cases of appendicitis (6–9).

Extended hospital stays impose additional strain on pediatric patients and their families and are associated with escalated healthcare expenses and utilization of medical resources. Furthermore, the duration of hospitalization serves as a significant indicator of resource consumption (10); predicting hospital stay length is crucial for optimizing resource allocation.

In recent years, several scoring systems have been developed for pediatric appendicitis, including the widely used Alvarado scores and Pediatric Appendicitis (PAS) scores (11, 12). However, these models primarily focus on diagnosis rather than predicting the length of stay (LOS). Previous studies (13–17) have shown that LOS in pediatric appendicitis is influenced by various factors, such as operative time, preoperative inflammatory markers, duration of symptoms, patient age, and interleukin-6 (IL-6). Despite this, most existing research has analyzed these predictors individually rather than integrating them into a comprehensive model. Furthermore, current predictive tools are predominantly based on Western populations, which may limit their applicability to Chinese pediatric patients. To address this gap, this study aimed to develop and validate a comprehensive nomogram that incorporates multiple variables to predict LOS in Chinese pediatric patients, thereby optimizing clinical decision-making and resource allocation.

## 2 Materials and methods

### 2.1 Patient selection

Research approval was obtained from the Ethics Committee of Suining Central Hospital. This is a retrospective study, and we collected information on children with appendicitis in the Department of Pediatric Surgery at Suining Central Hospital between January 2021 and December 2022.

The inclusion criteria for this study were as follows: individuals under the age of 18, of both sexes, with complete clinical data; all participants underwent laparoscopic appendectomy; and pathological examination confirmed acute appendicitis.

The exclusion criteria for this study are as follows: 1. congestive appendicitis confirmed by pathological examination; 2. negative appendectomy (normal appendix); 3. periappendiceal abscess (primarily managed conservatively with initial anti-infection or drainage treatment and subsequent laparoscopic appendectomy); 4. presence of other specific infectious diseases, such as lung infection; 5. during intraoperative exploration, various pathologies were identified including Merkel diverticulum, cholecystitis, pancreatitis, volvulus, oophoritis, adnexal torsion, torsion of ovarian cyst pedicle, rupture of corpus luteum cyst, acute pelvic inflammatory disease, and other conditions; 6. prior use of antibiotics or other medications before admission; 7. presence of concomitant blood system diseases, endocrine system diseases, or other severe medical conditions.

Acute appendicitis is classified into two categories: uncomplicated and complicated. Uncomplicated appendicitis refers to localized inflammation of the appendix without necrosis or perforation, whereas complicated appendicitis is defined by necrosis affecting part or all of the appendiceal wall, which may progress to perforation (18). According to the inclusion and exclusion criteria of this study, uncomplicated appendicitis corresponds to cases of suppurative appendicitis, while complicated appendicitis encompasses cases of gangrenous and perforated appendicitis.

### 2.2 Observation indicators

The primary outcome measure in this study was LOS, with prolonged LOS defined as admission for a duration equal to or exceeding the 75th percentile of LOS, including the discharge day (10, 19, 20). Additionally, data on patient demographics such as gender, age, height, weight, body mass index (BMI), onset time (from symptom onset to hospital admission), history of fever (>37.5℃ before admission), symptoms of emesis, diarrhea, peritoneal irritation, temperature, laboratory indexes upon admission, presence of appendiceal fecalith, drainage tube placement, and other relevant characteristics were collected. The presence of appendiceal fecalith was determined through multiple diagnostic modalities: preoperative imaging (CT/ultrasound), intraoperative findings, and pathological examination. A case was considered positive for fecalith if any of these methods confirmed its presence. Intraoperative drain placement was determined based on the following criteria: gangrenous or perforated appendicitis, significant purulent peritoneal fluid, extensive peritoneal adhesions, significant bleeding, or difficult dissection. All drains were surgical drains placed during the appendectomy procedure and were removed when drainage output was minimal and clear.

### 2.3 Statistical analysis

All statistical analyses were conducted using Empower software (version 4.2; http://www.empowerstats.com) and R software (version 4.4.0; https://www.R-project.org). The data in this study exhibited non-normal distribution, thus the Mann-Whitney U test (for two groups) and Kruskal-Wallis test (for three groups) were utilized for continuous variables, with results reported as median (interquartile range). Categorical variables were presented as frequencies (percentages) and analyzed using the chi-square test. Baseline characteristics were analyzed not only by LOS groups but also according to the histological subtypes of acute appendicitis (i.e., suppurative, gangrenous, and perforated).

Risk factor analysis was conducted through univariate and multivariate logistic regression analyses. Factors found to be statistically significant in the univariate analysis (*p* *<* 0.05) were further analyzed using the forward stepwise regression method to determine their inclusion in the final multivariable logistic regression model. Characteristics were presented as odds ratios (OR) with corresponding 95% confidence intervals (CI) and *p*-values. A two-sided significance level was used for all statistical tests.

A nomogram prediction model for prolonged LOS was developed using independent variables identified through multivariate logistic regression analyses. The discriminative performance of the logistic regression model was evaluated using Harrell's C-index with a 95% confidence interval, and an adjusted C-index was computed through bootstrapping validation with 1,000 resamples. Calibration curves were generated to assess the accuracy of the nomogram. Goodness-of-fit of the model was evaluated by Hosmer-Lemeshow test. Additionally, a decision curve analysis was conducted to determine the clinical utility of the nomogram by quantifying net benefits at various threshold probabilities. The net benefit was determined by subtracting the false positive rate from the true positive rate while also considering the relative harm of omitting interventions vs. the adverse effects of unnecessary interventions.

To assess the economic burden linked to prolonged LOS, we performed a cost analysis comparing medical expenses between the normal LOS and prolonged LOS groups. The analyzed cost categories included total hospitalization costs, anesthesia costs, surgery costs, and drug costs. All costs were presented in Chinese Yuan (CNY).

## 3 Results

### 3.1 Patients' characteristics

The study included a cohort of 415 pediatric patients diagnosed with acute appendicitis, comprising 122 females (29.40%) and 293 males (70.60%). Based on intraoperative findings and postoperative pathological analysis, the cohort consisted of 249 cases of acute suppurative appendicitis (60%), 64 cases of acute gangrenous appendicitis (15.42%), and 102 cases of acute perforated appendicitis (24.58%). Furthermore, the median LOS was recorded as 7 days, with the 25th and 75th percentiles being 6 and 9 days, respectively. Consequently, extended hospital stays were classified as exceeding 9 days. Children were categorized into either the normal LOS group or the prolonged LOS group based on whether their LOS was <9 days or ≥9 days, respectively. Table 1 presents the demographic and clinical data for both groups of patients.

**Table 1:** Baseline characteristics of participants between two groups.

	Normal LOS (<9 days; *n* = 290)	Prolonged LOS (≥9 days; *n* = 125)	*p*-value
Gender			0.953
Female	85 (29.31%)	37 (29.60%)	
Male	205 (70.69%)	88 (70.40%)	
Age (years)	8.00 (6.00–11.00)	6.00 (4.00–10.00)	<0.001
Height (cm)	133.00 (118.00–150.00)	122.00 (106.00–142.00)	<0.001
Weight (kg)	29.05 (21.57–41.00)	23.30 (17.50–35.80)	<0.001
BMI (Kg/m^2^)	16.38 (14.88–18.91)	15.87 (14.79–18.14)	0.215
Onset time (hours)	24.00 (11.25–24.00)	48.00 (24.00–72.00)	<0.001
History of fever			<0.001
No	198 (68.28%)	53 (42.40%)	
Yes	92 (31.72%)	72 (57.60%)	
Emesis			0.525
No	114 (39.31%)	45 (36.00%)	
Yes	176 (60.69%)	80 (64.00%)	
Diarrhea			0.101
No	273 (94.14%)	112 (89.60%)	
Yes	17 (5.86%)	13 (10.40%)	
Peritoneal irritation			<0.001
No	70 (24.14%)	11 (8.80%)	
Yes	220 (75.86%)	114 (91.20%)	
Temperature (>37.5℃)			
No	250 (86.21%)	98 (78.40%)	0.047
Yes	40 (13.79%)	27 (21.60%)	
WBC (×10^9^/L)	15.35 (11.90–18.20)	17.20 (13.60–21.80)	<0.001
Neutrophil count (×10^9^/L)	12.54 (9.26–15.41)	14.72 (10.69–18.46)	<0.001
Neutrophil ratio (%)	82.60 (76.20–87.77)	84.80 (78.40–89.20)	0.057
Lymphocyte count (×10^9^/L)	1.52 (1.07–2.15)	1.51 (0.91–2.17)	0.452
Lymphocyte ratio (%)	10.70 (6.82–16.05)	8.40 (5.40–14.30)	0.006
NLR	7.72 (4.82–12.88)	10.10 (5.48–16.09)	0.009
RBC (×10^9^/L)	4.54 (4.30–4.81)	4.59 (4.29–4.81)	0.906
Hemoglobin (g/L)	126.00 (120.00–132.75)	123.00 (116.00–130.00)	0.003
Platelet (×10^9^/L)	281.50 (231.00–331.00)	308.00 (254.00–375.00)	0.002
CRP (mg/L)	4.79 (0.00–29.67)	68.28 (18.94–123.10)	<0.001
ALT (U/L)	13.00 (10.70–17.00)	11.60 (9.10–16.60)	0.011
AST (U/L)	26.05 (22.00–30.00)	25.50 (20.00–30.10)	0.296
Direct bilirubin (umol/L)	2.90 (2.00–4.00)	2.80 (2.00–4.70)	0.431
Indirect bilirubin (umol/L)	6.70 (4.40–9.85)	6.10 (3.90–8.50)	0.301
Albumin (g/L)	47.20 (44.45–49.60)	44.00 (40.70–47.10)	<0.001
Operative time (minutes)	45.00 (40.00–55.00)	65.00 (55.00–85.00)	<0.001
Appendiceal fecalith			<0.001
No	171 (58.97%)	31 (24.80%)	
Yes	119 (41.03%)	94 (75.20%)	
Drainage tube			<0.001
No	175 (60.34%)	13 (10.40%)	
Yes	115 (39.66%)	112 (89.60%)	

BMI, body mass index; WBC, white cell count; NLR, neutrophil-to-lymphocyte ratio; RBC, red blood cell; CRP, c-reactive protein; ALT, alanine aminotransferase; AST, aspartate aminotransferase.

When further analyzing the characteristics among different histological subtypes (Table 2), significant differences were identified across the three groups. The LOS demonstrated a progressive increase from suppurative (7.00 days, IQR: 6.00–7.00) to gangrenous (8.00 days, IQR: 7.00–9.00) and perforated appendicitis (9.00 days, IQR: 8.00–12.00) (*p* < 0.001). Patients with perforated appendicitis were younger, had a longer onset time, and exhibited elevated inflammatory markers, including WBC, CRP, and NLR, compared to the suppurative and gangrenous groups (all *p* *<* 0.05). Appendiceal fecalith (80.39%) and the requirement for drainage tube placement (90.20%) were most frequent in perforated appendicitis, and operative time was the longest (median: 60.00 min, *p* < 0.001).

**Table 2:** Baseline characteristics of participants among histological subtypes of acute appendicitis.

	Suppurative AA (*n* = 249)	Gangrenous AA (*n* = 64)	Perforated AA (*n* = 102)	*p*-value
LOS (days)	7.00 (6.00–7.00)	8.00 (7.00–9.00)	9.00 (8.00–12.00)	<0.001
Gender				0.355
Female	73 (29.32%)	23 (35.94%)	26 (25.49%)	
Male	176 (70.68%)	41 (64.06%)	76 (74.51%)	
Age (years)	8.00 (6.00–11.00)	7.00 (4.00–11.00)	6.00 (4.00–9.75)	0.001
Height (cm)	135.00 (120.00–150.00)	127.50 (110.00–148.00)	120.00 (105.00–140.00)	<0.001
Weight (kg)	30.60 (22.00–41.30)	25.20 (18.82–38.25)	22.90 (17.50–32.88)	<0.001
BMI (kg/m^2^)	16.52 (14.96–19.23)	16.21 (14.88–18.29)	15.65 (14.56–17.59)	0.021
Onset time (hours)	20.00 (10.00–24.00)	24.00 (24.00–48.00)	48.00 (24.00–48.00)	<0.001
History of fever				<0.001
No	190 (76.31%)	30 (46.88%)	31 (30.39%)	
Yes	59 (23.69%)	34 (53.12%)	71 (69.61%)	
Emesis				0.175
No	104 (41.77%)	23 (35.94%)	32 (31.37%)	
Yes	145 (58.23%)	41 (64.06%)	70 (68.63%)	
Diarrhea				0.928
No	232 (93.17%)	59 (92.19%)	94 (92.16%)	
Yes	17 (6.83%)	5 (7.81%)	8 (7.84%)	
Peritoneal irritation				<0.001
No	66 (26.51%)	7 (10.94%)	8 (7.84%)	
Yes	183 (73.49%)	57 (89.06%)	94 (92.16%)	
Temperature (>37.5℃)				<0.001
No	228 (91.57%)	52 (81.25%)	68 (66.67%)	
Yes	21 (8.43%)	12 (18.75%)	34 (33.33%)	
WBC (×10^9^/L)	14.80 (11.70–18.00)	17.50 (14.65–22.05)	16.65 (12.80–20.38)	<0.001
Neutrophil count (×10^9^/L)	12.12 (8.84–15.16)	15.03 (11.52–18.85)	14.08 (10.46–17.51)	<0.001
Neutrophil ratio (%)	81.60 (74.80–87.40)	84.60 (79.97–88.43)	85.05 (80.60–89.55)	0.002
Lymphocyte count (×10^9^/L)	1.57 (1.09–2.22)	1.66 (1.05–2.11)	1.28 (0.83–2.01)	0.037
Lymphocyte ratio (%)	11.10 (6.80–16.80)	9.20 (6.02–13.50)	8.30 (5.62–11.65)	<0.001
NLR	7.41 (4.46–12.84)	9.28 (5.93–14.42)	10.47 (7.11–15.13)	0.001
RBC (×10^9^/L)	4.55 (4.32–4.79)	4.50 (4.23–4.86)	4.59 (4.29–4.84)	0.929
Hemoglobin (g/L)	126.00 (119.00–132.00)	124.00 (117.00–131.25)	124.50 (118.25–132.00)	0.352
Platelet (×10^9^/L)	281.00 (231.00–337.00)	287.50 (246.50–336.25)	306.50 (252.50–370.75)	0.020
CRP (mg/L)	3.03 (0.00–16.60)	41.88 (13.70–82.08)	59.40 (32.35–120.41)	<0.001
ALT (U/L)	13.20 (11.00–17.90)	11.85 (10.00–17.00)	11.00 (9.00–14.88)	<0.001
AST (U/L)	26.00 (21.80–30.00)	25.95 (21.10–32.00)	25.90 (21.00–28.90)	0.699
Direct bilirubin (umol/L)	2.70 (1.90–3.70)	3.20 (2.48–4.95)	3.40 (2.00–5.02)	<0.001
Indirect bilirubin (umol/L)	5.90 (4.00–8.50)	7.10 (5.40–10.70)	7.40 (4.25–10.40)	0.009
Albumin (g/L)	47.30 (44.60–49.70)	46.55 (42.48–48.52)	43.95 (41.32–46.50)	<0.001
Operative time (minutes)	45.00 (35.00–55.00)	55.00 (40.00–66.25)	60.00 (50.00–85.00)	<0.001
Appendiceal fecalith				<0.001
No	161 (64.66%)	21 (32.81%)	20 (19.61%)	
Yes	88 (35.34%)	43 (67.19%)	82 (80.39%)	
Drainage tube				<0.001
No	162 (65.06%)	16 (25.00%)	10 (9.80%)	
Yes	87 (34.94%)	48 (75.00%)	92 (90.20%)	

AA, acute appendicitis; LOS, length of stay; BMI, body mass index; WBC, white cell count; NLR, neutrophil-to-lymphocyte ratio; RBC, red blood cell; CRP, c-reactive protein; ALT, alanine aminotransferase; AST, aspartate aminotransferase.

Of the 415 participants, 164 underwent postoperative microbiological cultures, with positive results identified in 71 cases. The most frequently isolated pathogens were E. coli (*n* = 47) and Pseudomonas aeruginosa (*n* = 14). Participants in the prolonged LOS group exhibited a higher rate of positive cultures (45/125, 36%) compared to those in the normal LOS group (26/290, 9%).

### 3.2 Univariate and multivariate logistic regression analysis

Prolonged LOS served as the dependent variable throughout our analyses. Variables potentially correlated with prolonged LOS were identified through univariate analyses. Factors such as age, onset time, history of fever, peritoneal irritation, temperature (>37.5℃), white blood cell count (WBC), neutrophil count (neutrophil percentage in the total WBC), lymphocyte ratio (lymphocyte percentage in the total WBC), neutrophil-to-lymphocyte ratio (NLR), hemoglobin, platelet count, C-reactive protein level (CRP), albumin level, operative time, presence of appendiceal fecalith, and use of a drainage tube were found to be closely associated with prolonged LOS. Variables that were found to be statistically significant in the univariate analysis were included in the multivariate logistic regression, and some variables were removed using the forward stepwise regression approach. The final analysis revealed that age, NLR, CRP, operative time, appendiceal fecalith, and drainage tube were identified as independent risk factors for prolonged LOS. Further information can be found in Table 3 and Table 4.

**Table 3:** Univariable analyses of variables.

	OR (95%CI)	*p-*value
Gender(male)	0.99 (0.62, 1.56)	0.9526
Age (years)	0.87 (0.82, 0.93)	<0.0001
BMI	0.99 (0.93, 1.06)	0.7667
Onset time (hours)	1.03 (1.02, 1.04)	<0.0001
History of fever	2.92 (1.90, 4.51)	<0.0001
Emesis	1.15 (0.75, 1.78)	0.5247
Diarrhea	1.86 (0.88, 3.97)	0.1059
Peritoneal irritation	3.30 (1.68, 6.47)	0.0005
Temperature (>37.5℃)	1.72 (1.00, 2.96)	0.0490
WBC (×10^9^/L)	1.08 (1.04, 1.13)	<0.0001
Neutrophil count (×10^9^/L)	1.08 (1.04, 1.13)	0.0001
Neutrophil ratio (%)	1.02 (0.99, 1.04)	0.1281
Lymphocyte count (×10^9^/L)	0.99 (0.78, 1.25)	0.9299
Lymphocyte ratio (%)	0.97 (0.94, 1.00)	0.0223
NLR	1.04 (1.01, 1.07)	0.0046
RBC (×10^9^/L)	1.01 (0.62, 1.64)	0.9743
Hemoglobin (g/L)	0.98 (0.96, 0.99)	0.0052
Platelet (×10^9^/L)	1.00 (1.00, 1.01)	0.0004
CRP (mg/L)	1.02 (1.02, 1.03)	<0.0001
ALT (U/L)	0.99 (0.97, 1.01)	0.5260
AST (U/L)	1.00 (0.98, 1.03)	0.6910
Direct bilirubin (umol/L)	1.08 (0.99, 1.18)	0.0897
Indirect bilirubin (umol/L)	1.00 (0.95, 1.04)	0.8304
Albumin (g/L)	0.85 (0.81, 0.90)	<0.0001
Operative time (minutes)	1.04 (1.03, 1.06)	<0.0001
Appendiceal fecalith	4.36 (2.73, 6.96)	<0.0001
Drainage tube	13.11 (7.05, 24.39)	<0.0001

OR, odds ratio; CI, confidence interval; BMI, body mass index; WBC, white cell count; NLR, neutrophil-to-lymphocyte ratio; RBC, red blood cell; CRP, c-reactive protein; ALT, alanine aminotransferase; AST, aspartate aminotransferase.

**Table 4:** Multivariable logistic regression analysis of risk factors for prolonged LOS.

	OR (95%CI)	*p*-value
Age (years)	0.88 (0.81, 0.95)	0.0019
NLR	1.06 (1.02, 1.10)	0.0032
CRP (mg/L)	1.01 (1.01, 1.02)	<0.0001
Operative time (minutes)	1.02 (1.01, 1.04)	0.0002
Appendiceal fecalith	1.84 (1.03, 3.27)	0.0384
Drainage tube	4.11 (2.00, 8.45)	0.0001

The table presents results from the final multivariable logistic regression analysis, with prolonged LOS as the dependent variable. Variables included in the model were those found to be statistically significant (*p* < 0.05) in univariate analyses and retained after forward stepwise regression. Results are presented as adjusted OR with 95% CI and corresponding *p*-values.

LOS, length of stay; OR, odds ratio; CI, confidence interval; NLR, neutrophil-to-lymphocyte ratio; CRP, c-reactive protein.

### 3.3 The development of a predictive model

**Figure 1:**
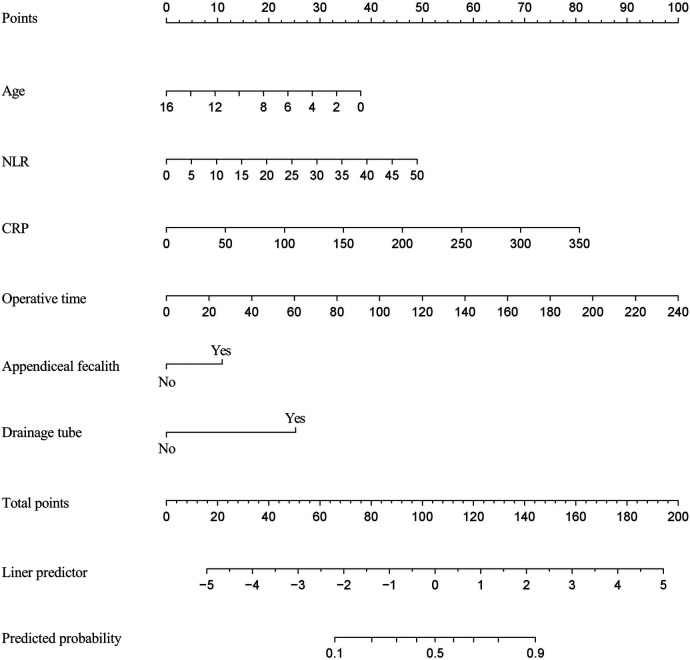
Nomogram to predict the probability of prolonged LOS. Various influencing factors are displayed in appropriate scales and the sum of all scores represents the probability of prolonged LOS.

Utilizing the independent predictors identified through multivariate logistic regression analysis, we developed an individualized nomogram prediction model for prolonged LOS, as depicted in Figure 1.

### 3.4 Discrimination and calibration

**Figure 2:**
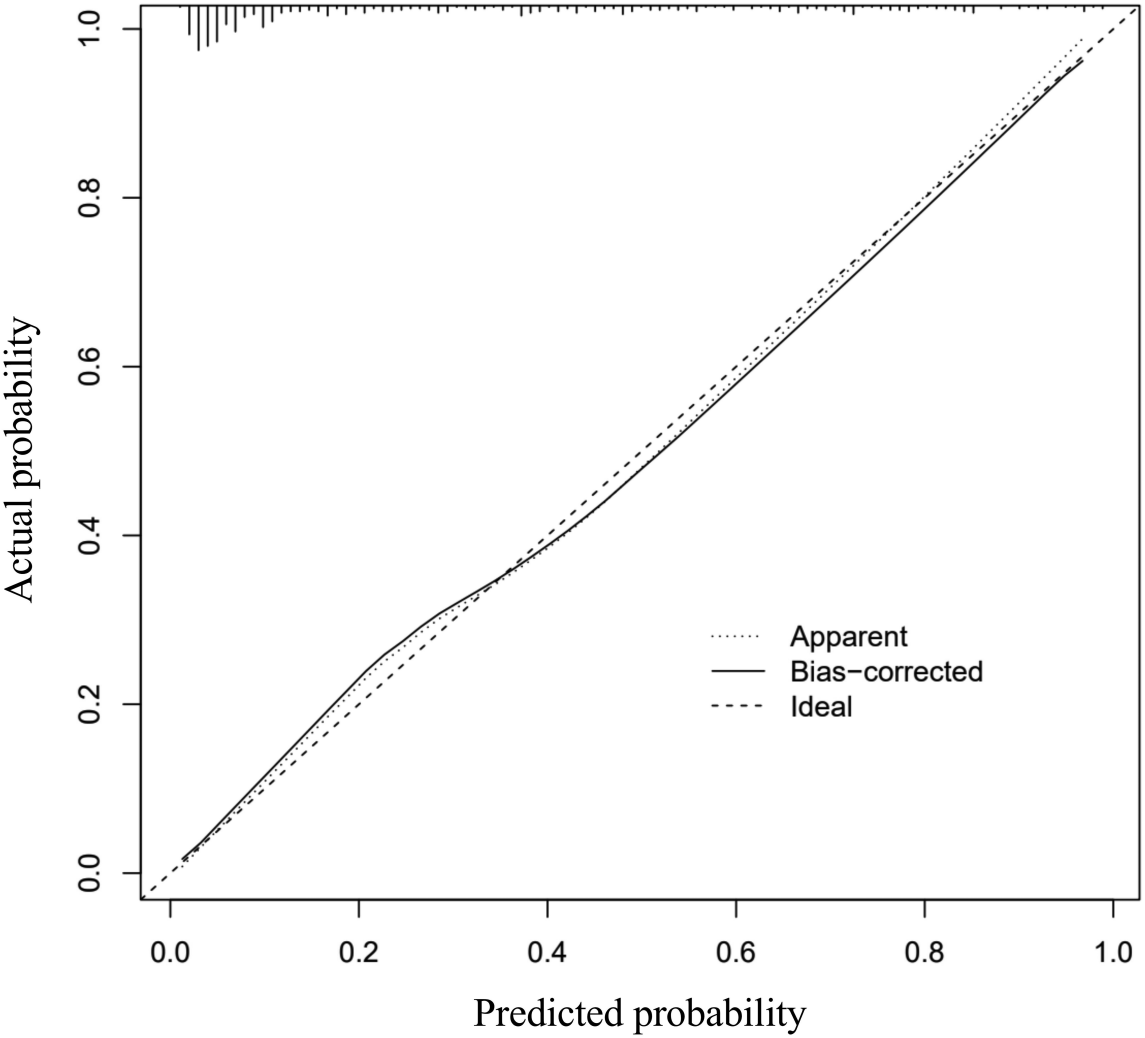
Calibration curves of the nomogram prediction. The *x*-axis is indicative of the nomogram's predictive value, while the *y*-axis represents the observed outcomes. The diagonal dotted line symbolizes the ideal model's perfect prediction, with the solid line denoting the nomogram's performance. A closer proximity of the solid line to the diagonal dotted line indicates a more accurate prediction by the nomogram.

The C-index of the prediction nomogram was determined to be 0.873 (95% CI: 0.838–0.908), with a corrected C-index of 0.8625 obtained through bootstrapping validation (1,000 resamples), indicating the model's favorable discrimination. Additionally, the Hosmer-Lemeshow fit goodness test *χ*^2^ = 4.7598, *p* = 0.7829, calibration curves illustrated a strong agreement between predicted and observed outcomes (Figure 2), further supporting the model's high predictive accuracy.

### 3.5 Clinical use

**Figure 3:**
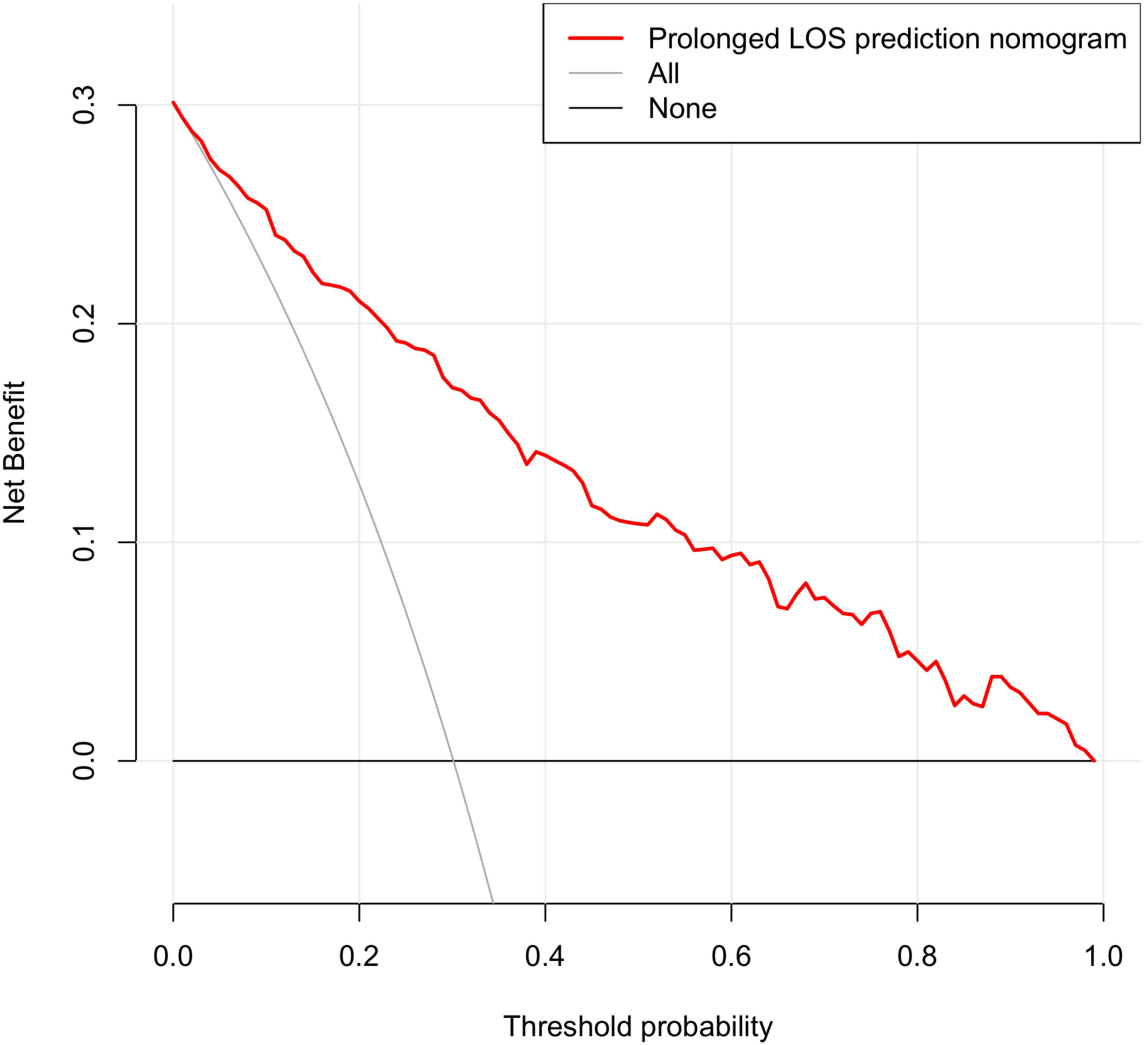
Decision curve analysis for the nomogram. The *x*-axis denotes the threshold probability, while the *y*-axis quantifies the net benefit. The red line represents the nomogram. The gray line represents the assumption that all cases had a prolonged length of stay. The black line represents the assumption that no cases had a prolonged length of stay. Utilizing this nomogram for predicting prolonged LOS can yield a net benefit when the threshold probability surpasses 2%.

According to the decision curve analysis of the nomogram, the predictive model demonstrates a net benefit at threshold probabilities exceeding 2% (Figure 3).

### 3.6 Cost analysis

As illustrated in Table 5, patients with prolonged LOS incurred significantly higher costs in all categories compared to those with normal LOS. Total hospitalization costs were approximately 28% higher in the prolonged LOS group. Among all cost categories, drug costs exhibited the largest disparity between the groups, followed by surgery costs and anesthesia costs (all *p* *<* 0.05).

**Table 5:** Comparison of medical costs between normal and prolonged LOS groups.

	Normal LOS (<9 days; *n* = 290)	Prolonged LOS (≥9 days; *n* = 125)	*p*-value
Total costs of hospitalization (CNY)	14,093.52 (12,667.27–15,480.20)	18,073.24 (16,170.34–20,459.71)	<0.001
Anesthesia costs (CNY)	952.00 (842.00–1,077.00)	990.00 (901.50–1,196.00)	0.017
Surgery costs (CNY)	2,757.00 (2,045.50–3,819.00)	3,311.00 (2,540.50–4,136.15)	<0.001
Drug costs (CNY)	1,871.52 (1,316.79–2,266.80)	2,962.99 (2,288.89–3,713.83)	<0.001

Values are presented as median (interquartile range). LOS, length of stay; CNY, Chinese Yuan.

## 4 Discussion

Reducing hospital stay duration for pediatric patients with appendicitis leads to decreased costs, increased bed utilization rates, and reduced wastage of medical resources. Therefore, investigating the factors influencing LOS is of paramount importance. This study developed a predictive model through the analysis of clinical data from children diagnosed with appendicitis. Findings indicated that factors such as age, neutrophil-to-lymphocyte ratio, C-reactive protein levels, operative duration, presence of appendiceal fecalith, and use of a drainage tube were significantly associated with prolonged LOS following laparoscopic appendectomy in children with appendicitis. These easily accessible predictors can assist surgeons in making informed decisions regarding resource allocation and facilitate improved communication with parents.

The results of our study indicate that younger age is correlated with an extended duration of hospitalization, possibly attributable to the presence of atypical clinical presentations in younger pediatric patients with appendicitis, leading to challenges in diagnosis and a higher severity of illness necessitating prolonged hospitalization for recovery. The high rates of peritoneal irritation observed in our study (75.86% in the normal LOS group vs. 91.20% in the prolonged LOS group, *p* < 0.001; and 73.49%, 89.06%, and 92.16% in suppurative, gangrenous, and perforated appendicitis, respectively, *p* < 0.001) require careful interpretation. In our pediatric cohort, peritoneal irritation was assessed through physical examination findings, including abdominal tenderness, rebound tenderness, and muscle guarding. However, these clinical signs are particularly challenging to assess in children, who often struggle to cooperate during examinations due to fear or anxiety. Although all examinations were performed by experienced pediatric surgeons, the inherent difficulties in examining children and the progressive nature of appendicitis (evidenced by longer onset times and higher inflammatory markers in more severe cases) may have influenced the reported rates. Future studies would benefit from incorporating more objective diagnostic criteria, particularly in pediatric populations where physical examination findings may be less reliable.

The NLR is a marker of systemic inflammatory response, reflecting the equilibrium between systemic inflammation and immune response (21, 22). Likewise, CRP functions as a non-specific indicator of inflammation, with elevated levels in plasma indicating infection and aiding in the clinical diagnosis of appendicitis (23–25). Elevated levels of NLR and CRP are indicative of heightened systemic inflammation (26–28). Furthermore, studies (17, 29–32) suggest that elevated NLR or CRP levels are associated with the development of complicated appendicitis and intra-abdominal abscesses.

Our research indicates a positive correlation between elevated levels of NLR and CRP with prolonged hospital stays. This association may be attributed to the inflammatory nature of NLR and CRP, which typically increase as appendicitis progresses. Elevated levels of NLR and CRP often signify severe infection, necessitating a longer recovery period post-surgery and extending the hospitalization duration. Additionally, our nomogram analysis reveals that CRP exhibits a stronger predictive capability for extended LOS compared to NLR as an inflammatory marker.

IL-6, a proinflammatory cytokine produced by various immune cells and tissue cells, serves as an early biomarker for tissue injury and systemic inflammation. It plays a crucial role in promoting the production of acute-phase proteins like CRP. Elevated levels of IL-6 have also been shown to assist in identifying bacterial infections (33, 34). Recent research (35) demonstrated that serum IL-6 is effective in distinguishing between complicated and uncomplicated cases of pediatric acute appendicitis. Moreover, studies (16) have indicated that appendicitis patients with a post-operative IL-6 increase exceeding 10% often experience prolonged hospitalization, potentially due to the association between elevated IL-6 levels and a heightened systemic inflammatory response. However, due to the data limitations of this retrospective study, IL-6 measurements were not included in our analysis. Nevertheless, we acknowledge the potential value of IL-6 in predicting post-operative inflammatory responses and clinical outcomes. Future research that integrates IL-6 assessment may further optimize predictive models and provide deeper insights into the role of systemic inflammation in recovery following pediatric appendicitis surgery, including its impact on hospitalization duration. This study found that longer surgeries are more likely to result in more extended hospital stays, aligning with the findings reported by Serres et al. (36), this relationship may be attributed to the association between prolonged operative time and the severity of intra-abdominal infection and adhesions. The presence of severe infection and adhesion often necessitates an expanded surgical intervention, leading to increased local exudation, formation of encapsulated effusion, impaired intestinal peristalsis, delayed recovery of gastrointestinal function, and, ultimately, an extended postoperative recovery period.

Appendicoliths have the potential to obstruct the appendiceal lumen, leading to bacterial overgrowth and continuous mucus secretion, resulting in lumen dilation and increased wall pressure, and this can trigger local inflammatory responses that may advance to gangrene and perforation. Moreover, research (37, 38) suggests that appendicoliths are an independent risk factor for appendiceal perforation, which can result in severe infection and prolonged recovery.

The routine use of abdominal drainage in cases of complicated appendicitis remains a topic of debate within the medical community (39). Proponents of this practice argue that abdominal drainage can effectively evacuate intra-abdominal collections, inhibit the accumulation of additional fluid, and facilitate the identification and drainage of fecal fistulas (40). However, in our investigation, children with appendicitis who underwent surgical placement of abdominal drainage exhibited over threefold increased odds of experiencing an extended hospitalization period, in contrast to children with appendicitis who did not undergo abdominal drainage. Prior research (41) has similarly demonstrated that abdominal drainage contributes to prolonged hospital stays in pediatric patients with appendicitis.

Theoretically, implementing drainage may lead to a decrease in surgical site infections and a reduction in the length of hospital stay. Nevertheless, emerging evidence (42) suggests that drainage may not effectively lower the occurrence of intra-abdominal abscesses and wound infections following complex appendicitis surgery. This phenomenon may be attributed to various factors. Potential causes include obstruction of the abdominal drainage tube, leading to diminished drainage efficacy, and the localized nature of abdominal drainage, which may result in incomplete drainage of the entire abdominal cavity, potentially leading to the development of peritoneal effusion and abscesses despite drainage efforts. Furthermore, the insertion of an abdominal drainage tube poses a potential risk for peritoneal or blood infection due to its direct placement into the abdomen, and this can also impede wound healing and elevate the likelihood of surgical site infection. Additionally, in pediatric patients, the presence of a drainage tube may contribute to a perception of illness, hindering postoperative recovery and ultimately prolonging the LOS. In addition, surgeons may opt for a more conservative approach in treating patients who have undergone drainage procedures, particularly those with a higher grade of acute appendicitis. This may involve postponing oral intake until the patient's abdominal condition improves, awaiting the removal of the abdominal drain, and potentially prolonging the hospital stay. Consequently, it is imperative to minimize the use of intraoperative drainage and promptly remove the drainage tube post-operation.

Our cost analysis revealed significantly higher expenses among patients with prolonged LOS across all cost categories. These results underscore the considerable economic burden of prolonged hospitalization, particularly for high-cost items such as drugs and surgeries. The prediction model proposed in this study has the potential to mitigate this issue. By identifying patients at risk of prolonged LOS early, the model could facilitate targeted interventions to minimize unnecessary delays, improve resource allocation, and reduce expenses. Although our analysis focused on direct costs, future research should investigate the broader economic impact, including indirect costs, to fully evaluate the model's cost-saving potential.

The present study is subject to several limitations. Firstly, the study population does not encompass all children with complicated appendicitis as periappendiceal abscesses were excluded. Secondly, the study excluded cases of congestive appendicitis, which represents the earliest and mildest phase of appendiceal inflammation. This decision was made to reduce heterogeneity within the study population and to concentrate on moderate-to-severe cases, which are more likely to result in prolonged hospital stays. However, this exclusion may introduce selection bias, thereby limiting the generalizability of our findings. Specifically, the predictive model developed in this study is primarily tailored to moderate-to-severe appendicitis (e.g., suppurative, gangrenous, and perforated appendicitis), leaving its applicability to milder cases uncertain. Future studies that include the full spectrum of appendicitis, including mild cases, could offer a more comprehensive understanding of the factors influencing LOS. Thirdly, the study utilized a retrospective design, introducing the possibility of biases such as information and selection bias. Additionally, as this study was conducted at a single center, internal validation was solely achieved through the bootstrap test, necessitating additional external validation utilizing data from multiple centers. Moreover, our study showed a male predominance, consistent with the known epidemiology of appendicitis (43). Although this reflects the natural disease pattern, the marked gender disparity may limit result generalizability. Future research should aim for more balanced gender distributions to enhance external validity. Furthermore, several clinically important variables, such as the extent of surgical contamination, postoperative abscess formation and the subsequent need for percutaneous/guided drainage, were not included due to incomplete documentation, which limited our ability to fully evaluate the relationship between intraoperative findings and postoperative outcomes. Finally, this study was limited by the high proportion of missing microbiological culture data (251/415 cases, 60.5%), which may introduce selection bias. The delayed availability of microbiological culture results (≥3 days) also limits their applicability to our aim of real-time LOS prediction. Future studies with more comprehensive datasets and an emphasis on the role of specific pathogens may provide greater insight into the relationship between infections and prolonged hospital stays.

In conclusion, we developed a nomogram with good accuracy to predict the length of hospital stay after laparoscopic appendectomy in children with appendicitis.

## Data Availability

The original contributions presented in the study are included in the article/Supplementary Material, further inquiries can be directed to the corresponding author.
